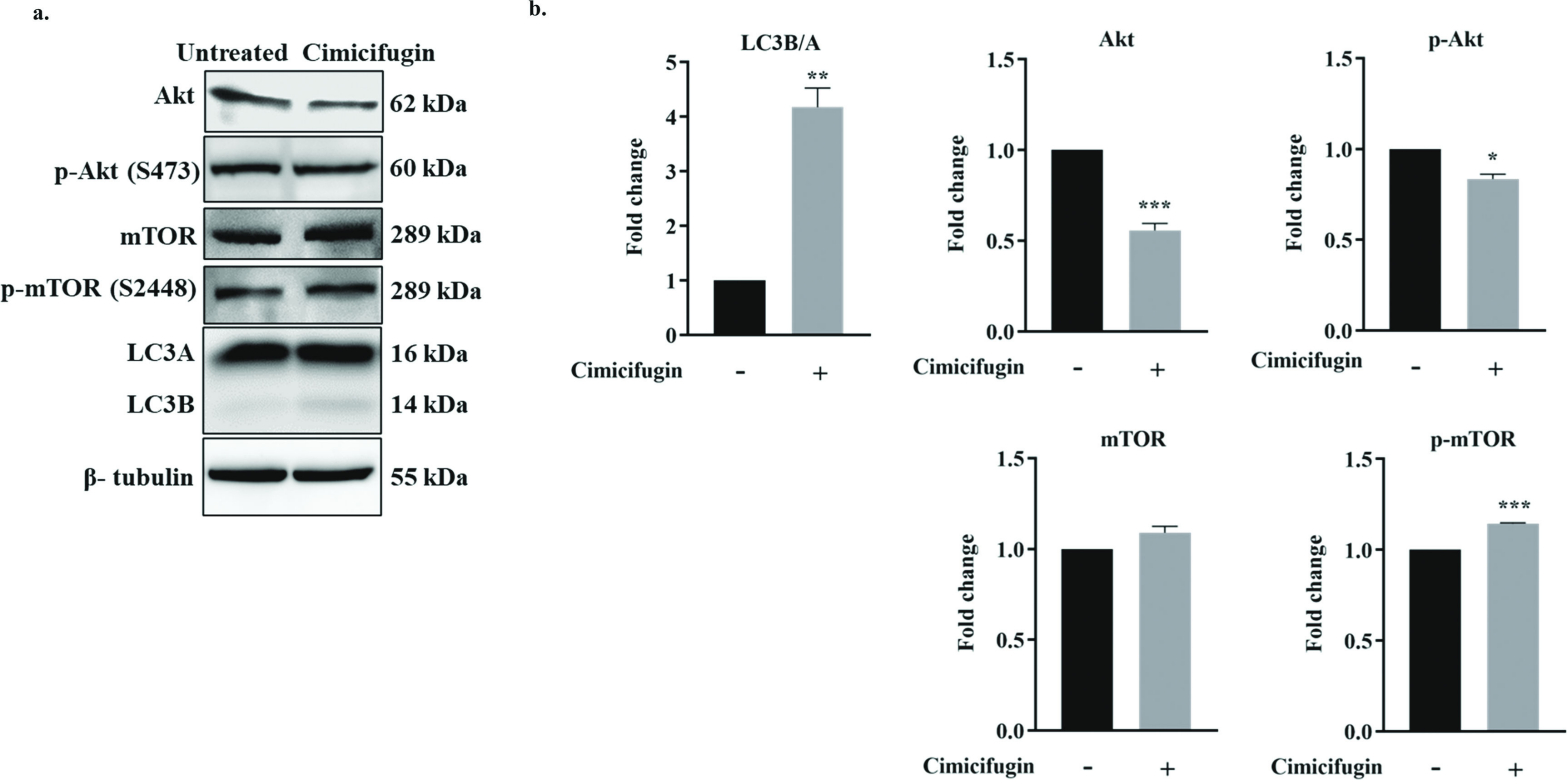# Erratum for Kar et al., “Cimicifugin, a broad-spectrum inhibitor of *Theileria annulata* and *Plasmodium falciparum* CDK7”

**DOI:** 10.1128/aac.01782-24

**Published:** 2025-01-24

**Authors:** Prajna Parimita Kar, Prasanna Babu Araveti, Kanika Saxena, Atlanta Borah, Puran Sijwali, Anand Srivastava

## ERRATUM

Volume 68, no. 8, e00440-24, 2024, https://doi.org/10.1128/aac.00440-24. Page 7: Figure 5 should appear as shown in this erratum. In the published article, Fig. 5 was a duplicate of Fig. 4. We apologize for this error, which did not change the final result.

**Fig 5:**